# Long-term toxicity of chlorpromazine, diclofenac and two lanthanides on three generations of *Ceriodaphnia dubia*

**DOI:** 10.7717/peerj.16472

**Published:** 2023-11-20

**Authors:** Brigita Gylytė, Viktoria Martinyuk, Reda Cimmperman, Rolandas Karitonas, Oksana Stoliar, Levonas Manusadžianas

**Affiliations:** 1 Nature Research Centre, Institute of Botany, Vilnius, Lithuania; 2 Ternopil Volodymyr Hnatiuk National Pedagogical University, Ternopil, Ukraine

**Keywords:** *Ceriodaphnia dubia*, Chlorpromazine, Diclofenac, Lanthanides, Gadolinium, Europium, Multigenerational toxicity

## Abstract

Amultigenerational study on *Ceriodaphnia dubia* was carried out by exposing three subsequent generations to pharmaceuticals chlorpromazine (CPZ) and diclofenac (DCF), and two lanthanide chlorides, gadolinium as GdCl_3_ and europium as EuCl_3_. As the treatments, environmentally relevant concentrations were chosen (0.001, 0.01 and 0.1 mg/L for CPZ; 0.1, 1 and 10 mg/L for DCF; 0.425, 4.25 and 42.5 µg/L for Gd and 0.41, 4.1 and 41 µg/L for Eu). Survival, population growth and reproduction success were evaluated at 21 and 30 days of exposure, and the whole observation period lasted 40 days. The least sensitive to all selected substances was the first daphnid generation (F1). Within 21-day exposure, no significant effects of the psychotropic drug CPZ on *C. dubia* survival were observed in generations F1–F3. The anti-inflammatory drug DCF did not affect survival in the F1 generation; however, it significantly reduced survival in the F3 generation at 1–10 mg/L. Both lanthanides did not affect survival in the F1 and F2 generations of *C. dubia* but considerably decreased survival in the F3 at 4–42 µg/L. Both pharmaceuticals stimulated the reproduction of *C. dubia* in the F1 generation, while inhibition occurred at the highest tested concentrations in generations F2 and F3. The inhibitory effect on the reproductive success of lanthanides in the F2 generation resembled that for CPZ but not for DCF. The dynamics of adverse effects during the 21–30-day period revealed that despite increased mortality in the controls (up to 30%), concentrations used in the study minified, in most instances, the survival and aggravated population growth and reproduction success of *C. dubia*. Our data suggest that *C. dubia* as a test organism can be used for 21 days in multigenerational investigations, especially when testing close to environmental concentrations. In this respect, the standard *C. dubia* chronic toxicity assay seems limited since prolonged observations and several generations of daphnids are required to obtain reliable information for the risk assessment of potentially aggressive chemicals.

## Introduction

It is well-known that pharmaceutical substances are currently found throughout the environment. For instance, the presence of pharmaceutical compounds in river and surface waters has frequently been reported (Sharma, Thakur & Kaushik, 2021). Concerning their ecotoxicity, some of them have been studied more extensively, *e.g.*, diclofenac (DCF) (Lonappan et al., 2016; Drzymała & Kalka, 2020), and others to a lesser extent, *e.g.*, chlorpromazine (CPZ) (Yuan et al., 2012; Adams et al., 2014). Both pharmaceuticals are among the 15 most hazardous substances in hospital effluents (HWW), and their hazard quotient exceeds 1000 (hazard quotient equals the ratio of the highest concentration ever measured in HWW and PNEC) (Frédéric & Yves, 2014). Diclofenac is a worldwide-used anti-inflammatory drug (Oliveira et al., 2015). DCF concentration in WW ranges globally within wide limits of 0.005–836 µg/L (see Sathishkumar et al., 2020). Chlorpromazine is the first-generation neuroleptic medication known as a dopamine D2 receptor antagonist (Li, Snyder & Vanover, 2016). CPZ was introduced into clinical practice in the middle of XX c. and is widely used as a low-cost psychotropic drug (Adams et al., 2014). The antiviral activity of CPZ has been recently reported, and the possibility of its usage against coronavirus infections is being discussed (Plaze et al., 2020; Stip et al., 2020). CPZ concentrations of 11.3 ng/L in sewage treatment plant effluent (Baresel et al., 2015) and 5–364 ng/L in psychiatric hospital WW (Yuan et al., 2012) have been measured. This pharmaceutical substance has been shown to cause toxicological effects on aquatic organisms such as protozoa (Nałęcz-Jawecki, Hajnas & Sawicki, 2008), micro- and macro-invertebrates (Oliveira et al., 2015; Alkimin et al., 2020; Khoma et al., 2022), macrophytes (Alkimin et al., 2019) and fish (Li et al., 2008).

Another group of emergent toxicants is rear earth elements (REEs) (Balaram, 2019). Gadolinium (Gd) occupies a special place among lanthanides (a 15-element group within REEs) because, in addition to its use in various modern technologies, it has been universally applied since the last decade of the 20th century as a contrast-enhancing element for magnetic resonance imaging (Rogowska, Ratajczyk & Wolska, 2018). To this end, global Gd use has increased almost 10-fold in a decade, from 20 million procedures in 1998 to 150–180 million in 2008 (Cyris, 2013). Based on the assumption that the diagnostic procedure requires 1.2 g of metal (Künnemeyer et al., 2009), approximately 180–220 tons of Gd is used per year, *i.e.,* 5% of annual Gd production. Diagnostic Gd complexes from the human body enter wastewater systems largely unmetabolised (Caravan et al., 1999). The natural background level of Gd in river waters has been estimated to be approximately 0.001–0.004 µg/L (Rogowska, Ratajczyk & Wolska, 2018). However, much higher levels of anthropogenic gadolinium are currently being recorded in European and global waters. According to Trapasso et al. (2021), Gd levels in rivers (Germany) were up to 0.18 µg/L (Bau & Dulski, 1996), in Ankara Stream (Turkey) 0.35 µg/L (Alkan, Alkan & Yanar, 2020), and in Orlando Easterly Wetlands (USA) up to 0.55 µg/L (Barber et al., 2015). Due to the increased amount of contrast agents, large amounts of Gd may remain bound in them, *e.g.*, in surface water samples from the Teltow canal near Berlin taken before and after the outlet of WWTP, the total Gd concentration increased significantly from 0.050 to 0.990 µg/L (Lindner et al., 2013). It has been pointed out that the highest pollution of Gd of anthropogenic origin is in the regions of a well-developed healthcare system (Rogowska, Ratajczyk & Wolska, 2018). Concerning another lanthanide, europium (Eu), which is envisaged to be applied for medical purposes, very little information on its effects on aquatic biota is available. This element has been classified as toxic to shrimps *Thamnocephalus platyurus* after 24-h exposure or as harmful to macrophytic algae of *Nitellopsis obtusa* after 24 days of exposure (Manusadžianas et al., 2020). Toxic effects of Eu on microalgae *Platymonas subcordiformis* and pearl oysters have also been reported (Ji et al., 2012). The concentration of Eu in surface waters (Poland) was measured to be 0.026 µg/L (Migaszewski & Gałuszka, 2016) and in effluents (Benin) 0.25 µg/L (Atinkpahoun et al., 2020).

Pharmaceuticals and REEs are measured in the aquatic environment at low concentrations in the ng/L–µg/L range. Short-term acute toxicity tests serve as informative tools for quantifying extreme pollution (Omotola et al., 2021); however, toxicity effects at low environmentally relevant concentrations can hardly be measured, and long-term exposures alongside sensitive endpoints are preferable. In this respect, multigenerational investigations with invertebrate organisms seem to be an essential source of ecotoxicological information since adverse effects may be visible in progeny. For instance, Dalla Bona et al. (2016) have demonstrated an inhibiting effect of enrofloxacin on reproduction in *Daphnia magna* in the second and third generations at environmentally relevant concentrations. Increased sensitivity of successive generations towards glucocorticoids has also been shown in *Ceriodaphnia dubia* (Bal et al., 2017). There are few studies of this kind on pharmaceuticals and not at all, to our knowledge, on lanthanides. Knowing that abnormally high levels of lanthanides enter surface waters, toxicological studies on representatives of different biological groups are needed. The majority of investigations with cladocerans have been conducted observing *D. magna*. This relatively large zooplankton species makes it vulnerable to fish predation and thus less ecologically representative, especially when considering lakes receiving pollution (Koivisto, 1995). Besides, although it has been reported that sensitivities of *D. magna* and *C. dubia* are generally comparable, there are exemptions showing higher sensitivity of *C. dubia* towards particular chemicals (Connors et al., 2022). Therefore, we focused on the long-term ecotoxicity of two classes of emergent pollutants, *i.e.,* pharmaceuticals (chlorpromazine and diclofenac) and lanthanides (gadolinium and europium), over three generations of *Ceriodaphnia dubia*. The effects induced by environmentally relevant concentrations of the substances were assessed on daphnids’ survival, population growth and reproduction success.

## Material and Methods

### Chemicals

Chlorpromazine hydrochloride [C_17_H_19_ClN_2_S ⋅HCl] (CPZ) and diclofenac sodium salt [C_14_H_10_Cl_2_NNaO_2_] (DCF) were purchased from Sigma-Aldrich (St. Louis, MO, USA). The purity of both chemicals was ≥98%. Stock solutions of 1 mg/L were prepared in deionised water, pH 7.5–7.6. Gadolinium (III) chloride hexahydrate [GdCl_3_ ⋅ 6H_2_O] (Gd) was purchased from Alfa Aesar Reacton (Kagel, Germany). Europium (III) chloride hexahydrate [EuCl_3_ ⋅ 6H_2_O] (Eu) was purchased from Aeros Organics (New Jersey, USA). Stock solutions of 0.1 mg/L were prepared in deionised water, pH 5.1 and 5.3, respectively. The purity of both chemicals was >99.9%.

Three nominal concentrations of each chemical were used: 0.001, 0.01 and 0.1 mg/L for CPZ; 0.1, 1and 10 mg/L for DCF; 0.425, 4.25 and 42.5 µg/L for Gd and 0.41, 4.1 and 41 µg/L 1 for Eu. The highest tested concentrations of Gd (425 µg/L) and Eu (410 µg/L) induced 100% mortality within two and four days of exposure, respectively.

### Culture conditions

Cladoceran *Ceriodaphnia dubia* cultures were started from dormant eggs (ephippia) obtained from MicroBioTests (Gent, Belgium). The tubes with ephippia were stored in the refrigerator at 5 °C before being used. The parental generation (F0) of *C. dubia* was hatched according to the bench protocol (SOP, 2020, Ceriodaphtoxkit F). The sensitivity of the test animals was checked with reference toxicant potassium dichromate (24-h EC50 = 0.4 mg/L, the value that fell into a diapason indicated in the specification sheet; EC50—an effective concentration that induces 50% immobilization of *C.  dubia*). The first brood of F0 was observed after 5–7 days. Only the neonates of daphnids that had ≥6 neonates were used to start the F1 generation culture.

Daphnids were cultured in 500 mL synthetic medium (reconstituted water) produced following ISO 20665:2008. The medium was composed of 1.2 g MgSO_4_, 1.92 g NaHCO_3_, 0.08 g KCl, 1.2 g CaSO_4_ ⋅ 2H_2_O and 0.04 mg Na_2_SeO_4_ dissolved in 20 L of deionised water. The medium was aerated for at least 48 h. Vitamin B12 (1–2 µg/L) was added to the medium before use. The reconstituted water (pH 7.4–7.8; 8.2–8.8 mgO_2_/L) was partially renewed weekly. The organisms were fed three times a week on microalgae suspension (2.5–3.5 ⋅ 10^7^ cells/mL of *Pseudokirchneriella subcapitata*, a culture maintained in the Laboratory of Aquatic Ecotoxicology) and Fish food Sera micron (5 mg/mL) (Sera, Germany).

### Life-cycle experiments

The experiments with *C. dubia* were performed on three successive generations following a modified protocol derived from the standardised one-generation-reproduction bioassay according to ISO 20665:2008. The experimentation took place between October and December 2021.

A single organism obtained from the stock culture was placed in every well of a 30-well plastic plate. Each well contained 10 mL of the test solution and 0.06 mL of food. The treatments consisted of 10 replicates (excluding the control group of 30 replicates). Tests were initiated with <48 h old neonates. This was the first generation of organisms (F1). Test solutions were renewed three times a week by preparing new replicates and transferring the adult daphnid from the old to the new test solution. The mortality of adult daphnids and the number of neonates produced by each of them were counted at this time. After the transfer of adult daphnids, each well was supplemented by 0.04 mL of concentrated algae and 0.2 mL of fish food. The plates were incubated at 25 ± 1 °C under 800 lx light with a 16: 8 (light: dark) cycle. Tests were considered complete after 40 d.

The second generation (F2) was composed of neonates produced by the F1 generation after 14–18 days. Collected neonates from the 4th–5th brood from control and each treatment were mixed and randomly distributed to produce the next generation. The procedure was repeated for a successive generation.

To reflect distinct aspects of multigenerational reproduction, two parameters were calculated at each generation, *i.e.,* population growth expressed as the mean of the offspring number per female daphnid and reproduction success expressed as the mean of the offspring number per surviving mother daphnid, both at the end of 21- and 30 days of exposure.

### Statistical analysis

The obtained data were based on one independent experiment with ten treatment replicates. The control consisted of 30 replicates (organisms). We used the exact Fisher test at *α* = 0.05 to compare the quantal (mortality probabilities) effects of two single concentrations at a particular time. To evaluate the variation of population growth and reproduction success, the data obtained in every generation after treatment with three concentrations and the control were analysed for distribution normality and homogeneity of variance using Shapiro–Wilk and Levene’s tests, respectively. Since the normality condition was met in each group, a one-way ANOVA followed by the *post hoc* Dunnett test (equal variances) or the Dunnett T3 (unequal variances) were applied; otherwise, the Kruskal-Wallis one-way ANOVA was applied. The analysis was carried out using the software PASW Statistics 18.0 (Predictive Analytics Software; IBM, Armonk, NY, USA).

## Results

**Figure 1:**
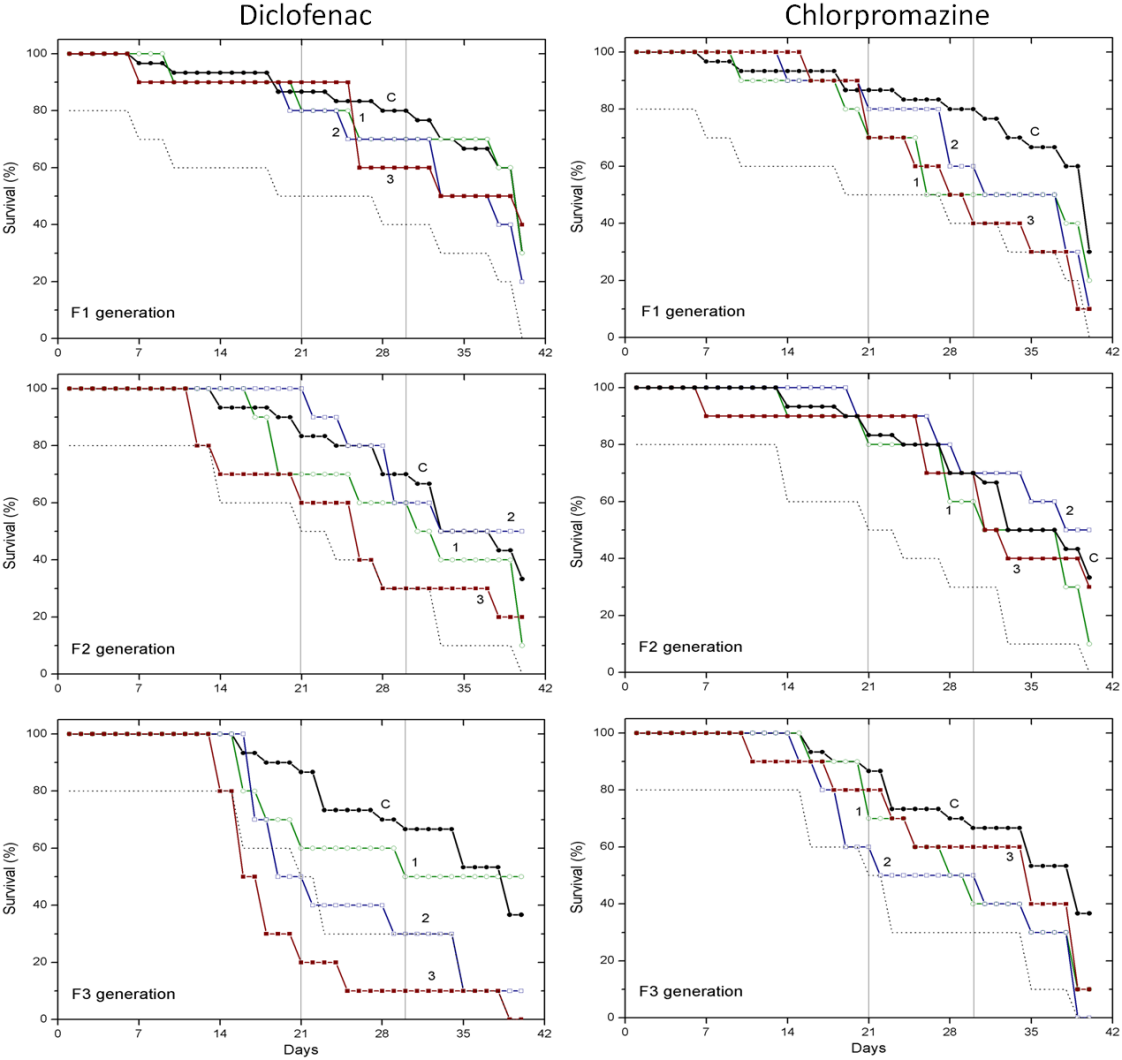
The effect of chlorpromazine and diclofenac on the survival of *C. dubia*. Survival of three successive generations of *Ceriodaphnia dubia* (F1, F2 and F3) exposed for 40 days in chlorpromazine: (C) control, (1) 0.001, (2) 0.01 and (3) 0.1 mg/L; or in diclofenac: (C) control, (1) 0.1, (2) 1.0 and (3) 10 mg/L. Vertical lines indicate survival percentage at days 21 and 30; the dotted line represents a statistically significant difference from controls at *α* = 0.05.

**Figure 2:**
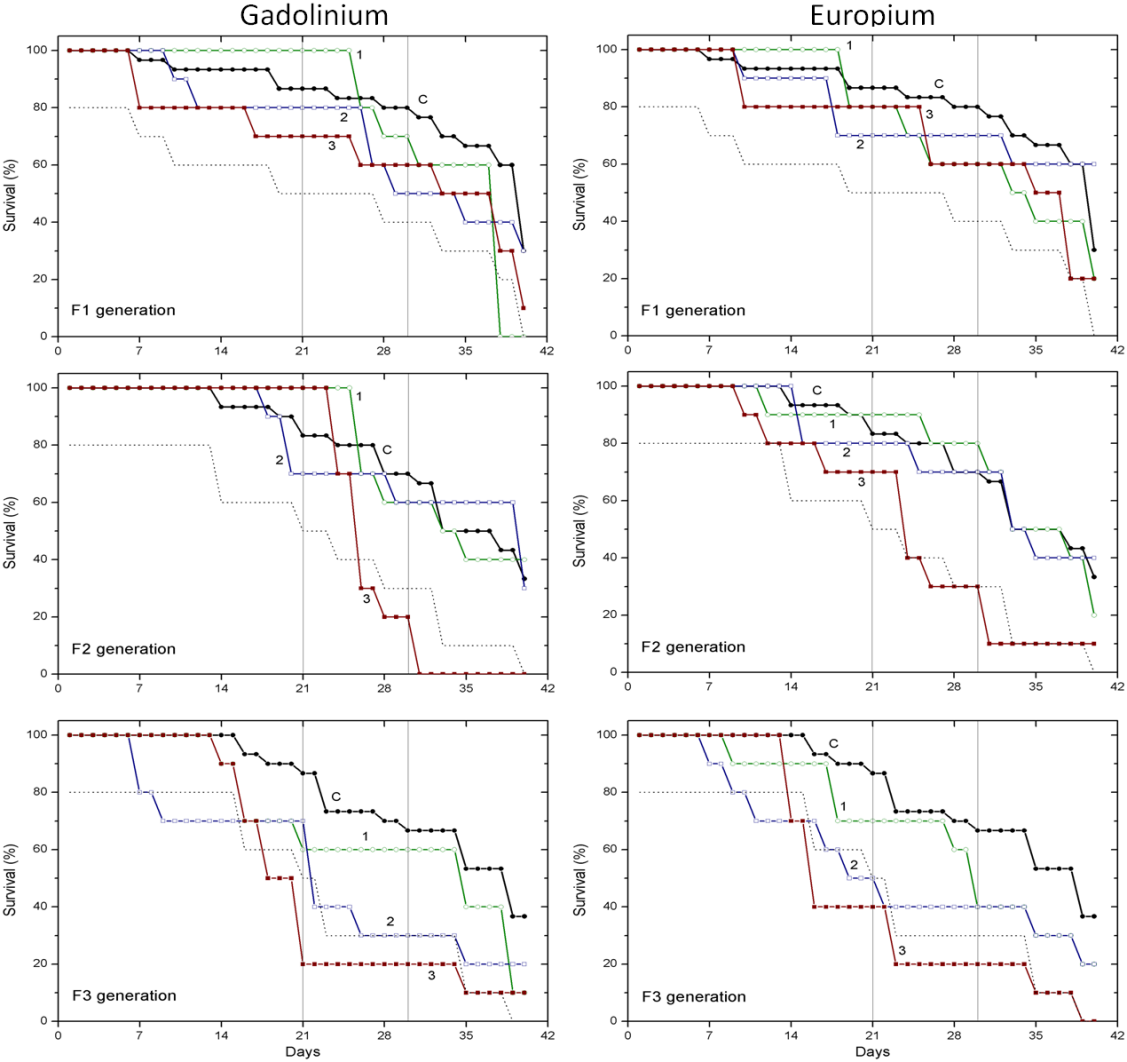
The effect of gadolinium and europium on the survival of *C. dubia*. Survival of three successive generations of *Ceriodaphnia dubia* (F1, F2 and F3) exposed for 40 days in GdCl_3: (C) control, (1) 0.425, (2) 4.25 and (3) 42.5 µgGd/L; or in EuCl_3: (C) control, (1) 0.41, (2) 4.1 and (3) 41 µgEu/L. Vertical lines indicate survival percentage at days 21 and 30; the dotted line represents a statistically significant difference from controls at *α* = 0.05.

Survival data obtained with two pharmaceuticals (chlorpromazine and diclofenac) and two lanthanides (gadolinium and europium) are presented in Figs. 1 and 2, respectively. Mortality of *C. dubia* in controls (C) throughout experimentation with daphnids of F1, F2 and F3 generations did not exceed 20% after 21-day exposure, 30% after 30-day exposure, and 60–70% on day 40.

The effect of CPZ on the survival of daphnids did not statistically differ from controls in F1–F3 generations at all tested concentrations (0.001, 0.01 and 0.1 mg/L) within 40-day exposures (Fig. 1). The effect of DCF did not statistically differ from the control in F1–F2 generations at all tested concentrations (0.1, 1 and 10 mg/L) within 40-day exposures, however, the survival was statistically significantly lower from controls (*α* = 0.05) in the F3 generation at 10 mg/L DCF. The survival began decreasing on day 14, reaching a 20% level on day 21. The effect observed at a ten-fold lower concentration showed up later on day 17, equalling 50% on day 19, a statistically significant level from the control (Fig. 1).

The effect of Gd and Eu on the survival of daphnids did not statistically differ from controls in the F1 generation at all tested concentrations (0.425, 4.25 and 42.5 µg/L for Gd and 0.41, 4.1 and 41 µg/L for Eu) within 40-day exposures (Fig. 2). In the F2 generation, the highest concentration of both lanthanides diminished the survival statistically significantly from controls (*α* = 0.05) starting from days 24–25 and was ≤40%. In the F3 generation, the highest concentration of both lanthanides diminished the survival statistically significantly from controls (*α* = 0.05) starting from days 14–18, reaching a survival rate of 20% on days 21–23. The lower, 4.25 and 4.1 µg/L concentrations of Gd and Eu, respectively, induced a weaker effect on survival rate reaching 40% on day 22 of exposure (Fig. 2).

**Figure 3:**
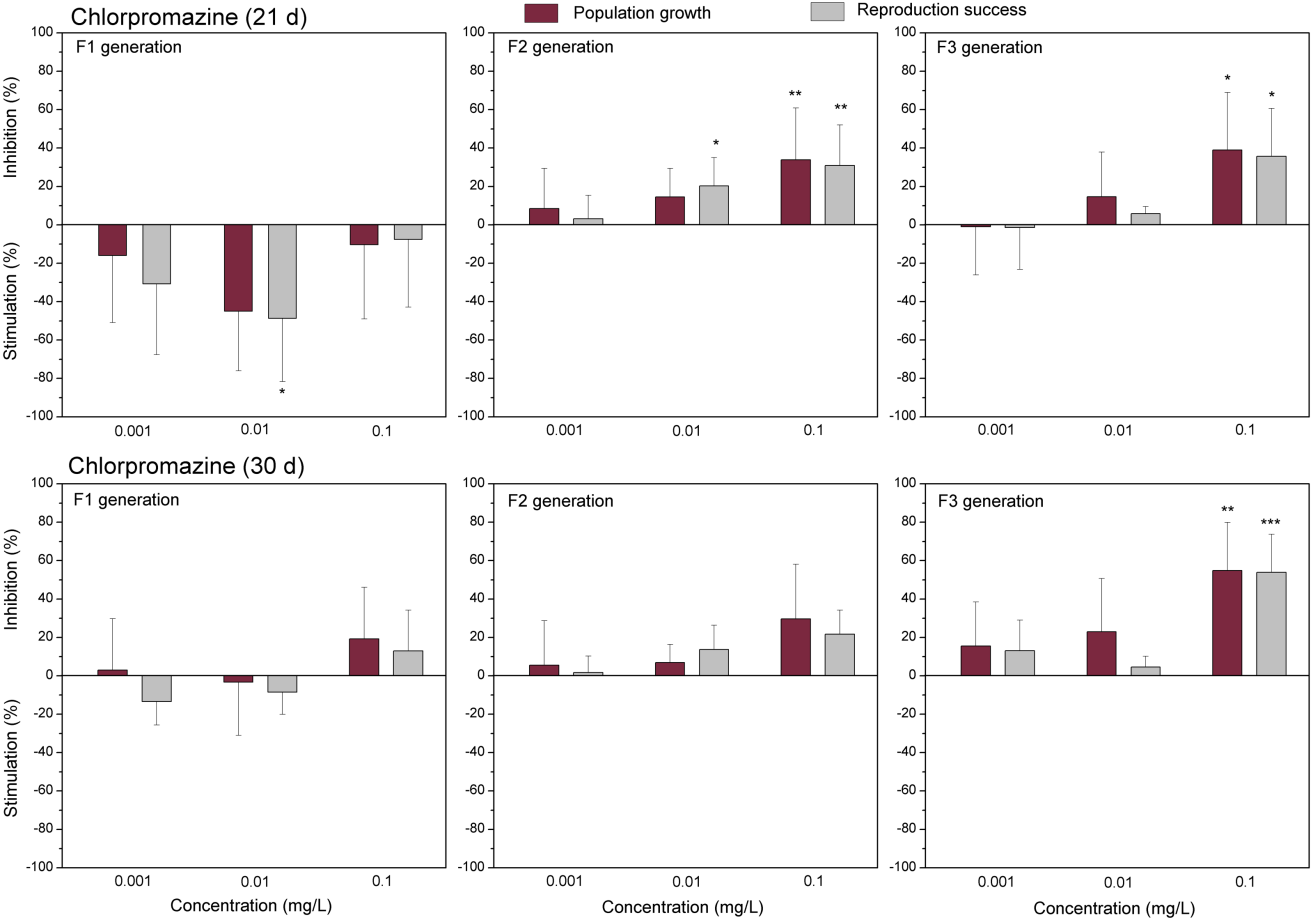
The effect of chlorpromazine on *C. dubia* reproduction parameters. The effect of chlorpromazine (0.001, 0.01and 0.1 mg/L) on population growth and reproduction success of three successive generations at 21 and 30 days of exposure. Impact on population growth is expressed as the mean variation percentage compared to a control of 10 replicates ± SD. Effect on reproduction success is expressed as the mean of variation percentage compared to control for the number of surviving mothers daphnids ± SD. The dotted lines represent significance limits of 30%. Asterisks indicate a statistically significant difference from the control: (*)*p* < 0.05, (**)*p* < 0.01, (***)*p* < 0.001.

**Figure 4:**
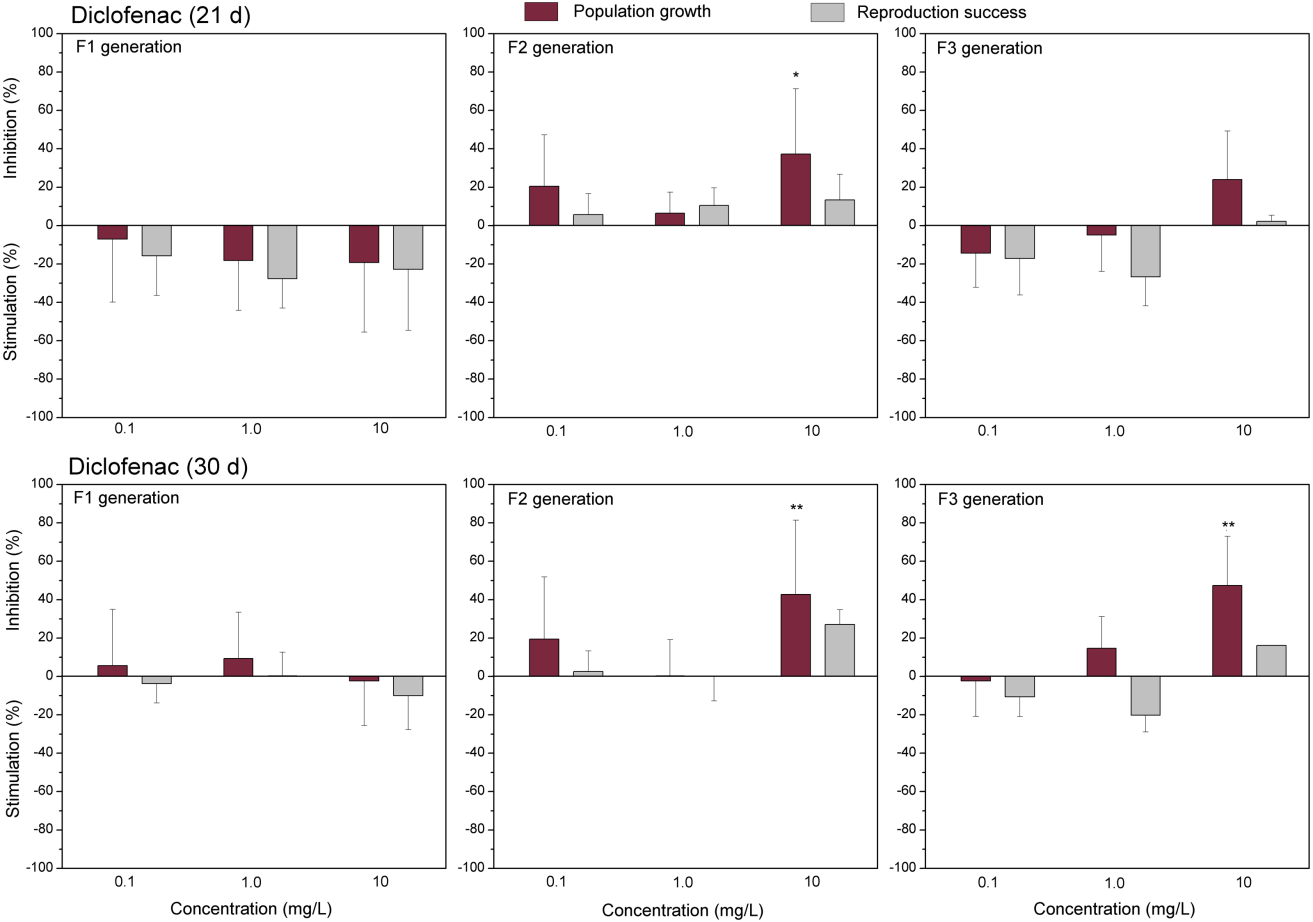
The effect of diclofenac on *C. dubia* reproduction parameters. The effect of diclofenac (0.1, 1.0 and 10 mg/L) on population growth and reproduction success of three successive generations at 21 and 30 days of exposure. Impact on population growth is expressed as the mean variation percentage compared to a control of 10 replicates ± SD. Effect on reproduction success is expressed as the mean of variation percentage compared to control for the number of surviving mothers daphnids ± SD. The dotted lines represent significance limits of 30%. Asterisks indicate a statistically significant difference from the control: (*)*p* < 0.05, (**)*p* < 0.01, (***)*p* < 0.001.

**Figure 5:**
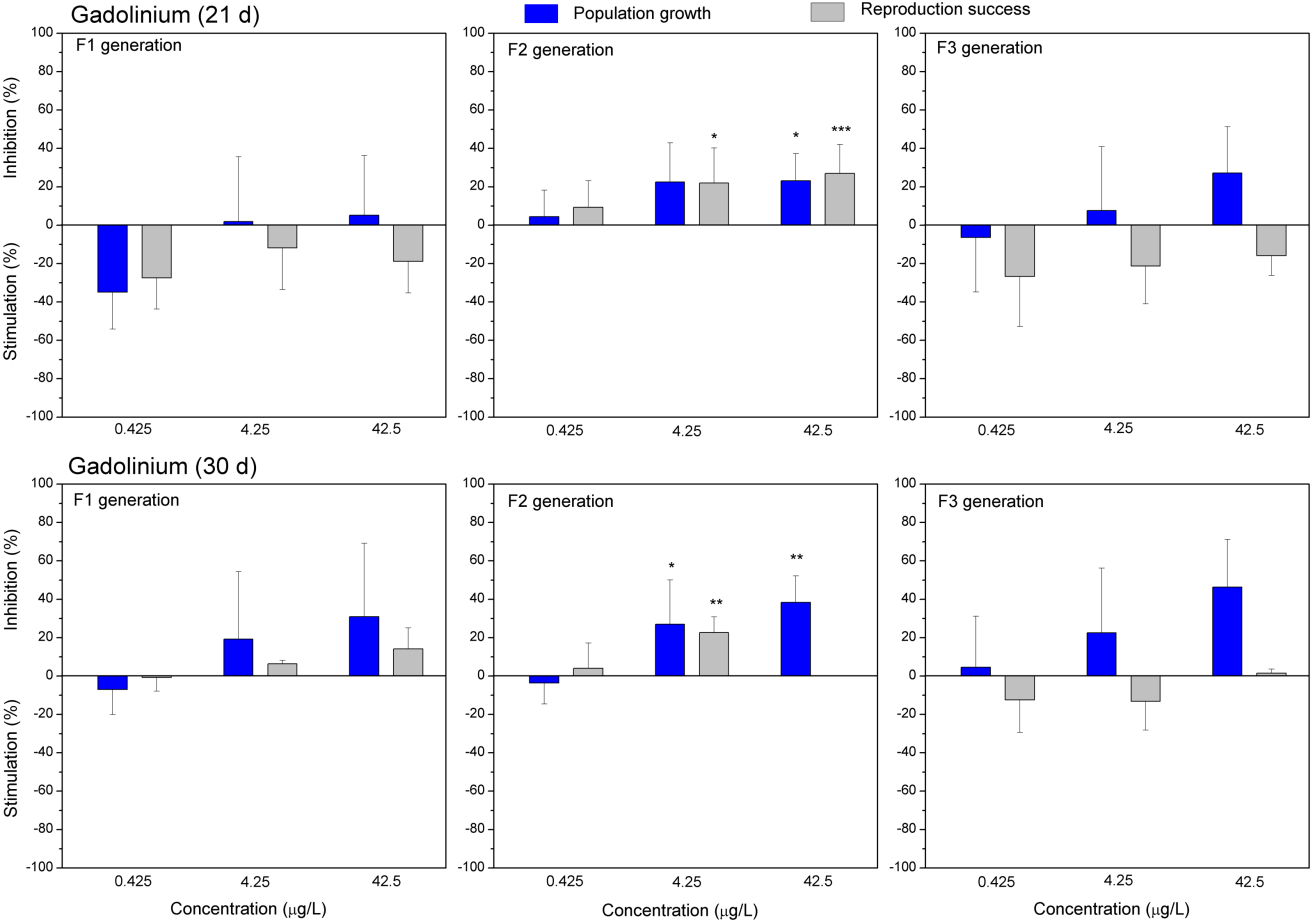
The effect of gadolinium on *C. dubia* reproduction parameters. The effect of GdCl_3_ (0.425, 4.25 and 42.5 µgGd/L) on population growth and reproduction success of three successive generations at 21 and 30 days of exposure. Impact on population growth is expressed as the mean variation percentage compared to a control of 10 replicates ± SD. Effect on reproduction success is expressed as the mean of variation percentage compared to control for the number of surviving mothers daphnids ± SD. The dotted lines represent significance limits of 30%. Asterisks indicate a statistically significant difference from the control: (*)*p* < 0.05, (**)*p* < 0.01, (***)*p* < 0.001.

**Figure 6:**
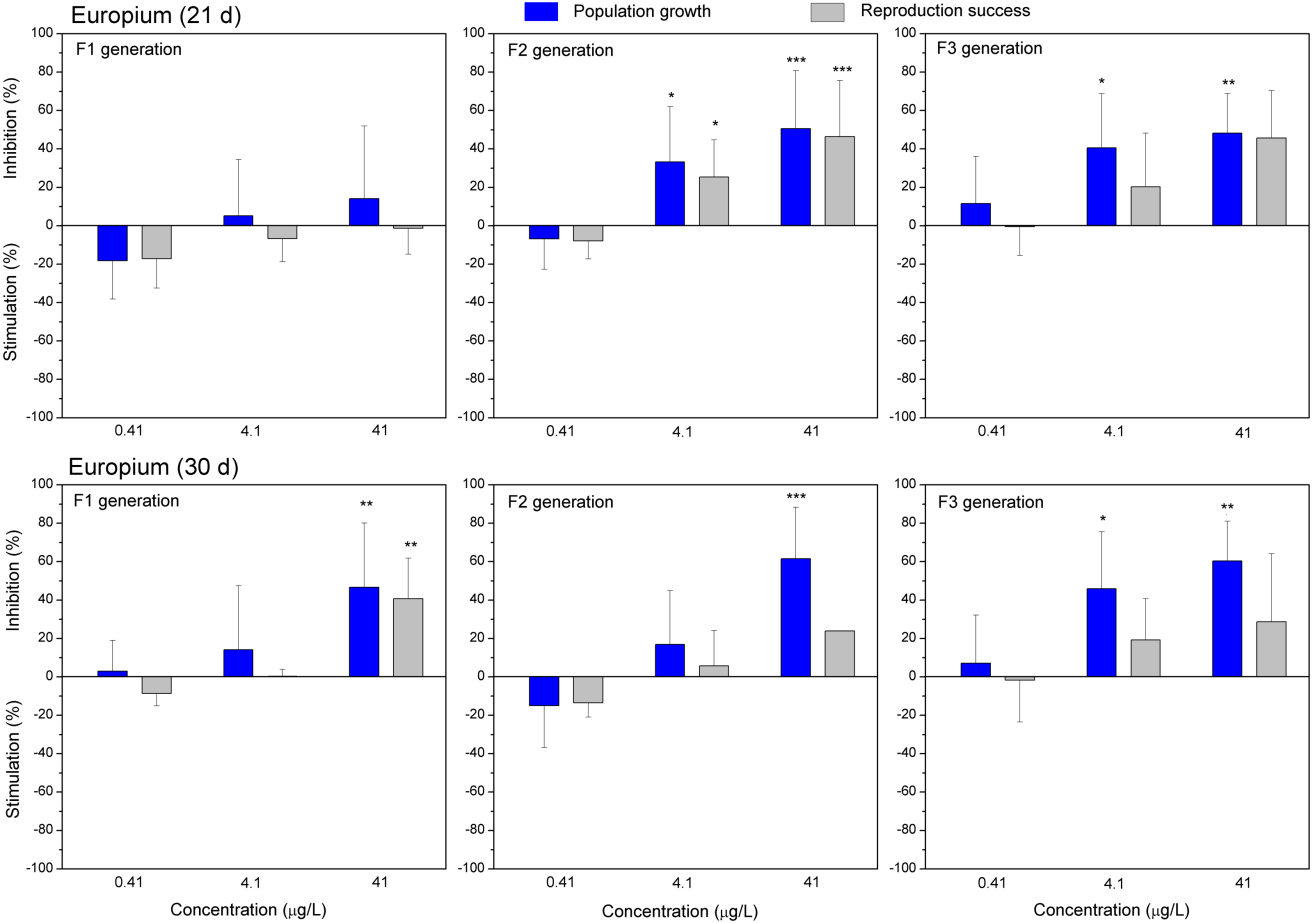
The effect of europium on *C. dubia* reproduction parameters. The effect of EuCl_3_ (0.41, 4.1 and 41 µgEu/L) on population growth and reproduction success of three successive generations at 21 and 30 days of exposure. Impact on population growth is expressed as the mean variation percentage compared to a control of 10 replicates ± SD. Effect on reproduction success is expressed as the mean of variation percentage compared to control for the number of surviving mothers daphnids ± SD. The dotted lines represent significance limits of 30%. Asterisks indicate a statistically significant difference from the control: (*)*p* < 0.05, (**)*p* < 0.01, (***)*p* < 0.001.

The data of studied chemical agents on population growth and reproductive success of *C. dubia* are presented in Figs. 3 and 4 (pharmaceuticals) and Figs. 5 and 6 (lanthanides). Both parameters (the population growth taking into account the total number of neonates per mother during the successive broods at each generation and the reproduction success taking into account the number of neonates per alive mother at each generation) did not show statistically significant inhibition in the F1 generation at 21- or 30-day of daphnid exposure to CPZ (Fig. 3). Instead, CPZ stimulated population growth and reproductive success in a concentration-dependent manner reaching a maximum (>40%) at 0.01 mg/L on day 21; however, no significant stimulation observed within 30 days of exposure at all tested concentrations (0.001–0.1 mg/L). In generation F2, population growth and reproductive success inhibition augmented with the increased CPZ concentration, reaching ≥30% inhibition relative to the control (*p* < 0.01) at the highest concentration on day 21. A similar tendency was observed on day 30 as well. In generation F3, both parameters reached the highest values at the highest CPZ concentration (0.1 mg/L), showing >30% inhibition on day 21 (*p* < 0.05) and >50% inhibition on day 30 (*p* < 0.01) (Fig. 3).

Similarly, as CPZ, the DCF showed 20–30% (not statistically significant) stimulation of population growth and reproductive success in generation F1 on day 21 and no effect on day 30 (Fig. 4). In generation F2, inhibition of population growth by 30–40% (*p* < 0.05) observed at the highest tested concentration (10 mg/L), on days 21 and 30. Under the same exposure conditions, reproductive success was less susceptible, showing inhibition by 15–25% (not statistically significant). In generation F3, the population growth was inhibited by 20% on day 21 and almost 50% on day 30 (*p* < 0.01) at the highest DCF concentration. No inhibition of reproductive success was observed at 0.1–10 mg/L DCF, rather a minor (not statistically significant) stimulation (10–25%) at 0.1–1 mg/L DCF (Fig. 4).

Both lanthanides, Gd (Fig. 5) and Eu (Fig. 6) induced (not statistically significantly) 20–30% stimulation of population growth and reproductive success of daphnids in generation F1 on day 21 at the lowest concentration (0.4 µg/L) and showed negligible reaction at higher concentrations (4–42 µg/L). The most visible alterations of the parameters (*p* < 0.01) in the F1 were observed for Eu on day 30, reaching ≥40% inhibition of population growth and reproductive success at the highest concentration (Fig. 6). In generation F2, statistically significant inhibitions of both parameters in the treatments with Gd (20–30%, Fig. 5) and Eu (25–50%, Fig. 6) were observed already on day 21 (*p* < 0.001–0.05), at both highest tested concentrations of each lanthanide. In generation F3, the population growth of daphnids decreased at both highest tested concentrations of each lanthanide, showing statistically significant inhibition (40–60% respective to controls, *p* < 0.01–0.05) in the case of Eu on days 21 and 30 (Fig. 6). At these concentrations, various effects of lanthanides on reproduction success observed in the F3. In the case of Eu, inhibition of this parameter reached 20–40%, while in the case of Gd, a minor stimulation came in sight on day 21 (Fig. 5).

## Discussion

To learn how pharmaceuticals and lanthanoids affect the overall longevity and reproduction of *Ceriodaphnia dubia*, we conducted a long-term multigenerational study over three generations. At least 50% of daphnids in the control groups survived for 35 days under the study’s experimental conditions. It has been previously reported that half of *C. dubia* has survived 24–32 days, depending on the composition of the media (Stewart & Konetsky, 1998). Additionally, the lifespan of *C. dubia* depends on the diet and type of feeding and ranges from 12 to 62 days at 24–25 °C (Cowgill, Takahashi & Landenberger, 1985; Stewart & Konetsky, 1998; Muñoz Mejía & Martínez-Jerónimo, 2007). On the 40th day of the observation, the percentage of surviving daphnids in the control groups was about 30% in all three generations. The first brood of *C. dubia* occurred after 5–10 days, which contradicts the overall duration of the test, *i.e.,* 7–8 days, according to the ISO 20665 standard. The requirement of at least three broods for ≥60% of females at the end of exposure was achieved on days 21 and 30 based on the neonate counting 10 days before days 21 and 30 (Supplementary Material). The lag period before the first brood could be associated with the volume of the testing chamber (10 mL instead of the least recommended volume of 15 mL) and/or feeding diet (Versteeg et al., 1997; Gosset et al., 2018). Other requirements of the ISO standard for *C. dubia* in the control medium, *i.e.,* mortality ≤20%, males ≤10%, and at least 15 neonates per alive female were fulfilled or close to these at days 21 and 30.

To date, no data on the effect of chlorpromazine, an antipsychotic drug, on *C. dubia* have been found in the referenced literature (Wronski & Brooks, 2023). Concerning other daphnia species, it has been reported that the total number of neonates per female throughout the 21 days of *D. magna* exposure at 0.01mg/L CPZ did not differ from the controls; meanwhile, time for a first brood increased (Alkimin et al., 2020). In contrast, we observed the stimulation effect of CPZ on population growth (corresponding parameter to a total number of neonates) and reproductive success of *C. dubia* on day 21 in the F1 generation at the lowest tested CPZ concentrations (0.001–0.01mg/L). Also, there was no significant effect on the time for the first brood (Supplementary Material). Interestingly, the observed stimulation of *C. dubia* population growth and reproduction success by CPZ on day 21 was absent on day 30, and this effect at the lowest tested concentrations (0.001–0.01mg/L) was evident only in the F1 generation. The total number of *D. magna* neonates has been reported to diminish at 0.25 mg/L CPZ (Oliveira et al., 2015), while we observed the effect on *C. dubia* at lower 0.1 mg/L concentration in the F2 and F3 generations but not in the F1 generation. Generally, the detrimental effects of contaminants may either increase or decrease across generations or remain unchanged (Campos et al., 2016). It is also a widely accepted opinion that neonates born from unexposed mothers are less susceptible to toxicants than those from exposed ones (Campos et al., 2016; Castro et al., 2018). Indeed, analysing the multigenerational effects of a set of toxicants, Barata et al. (2017) have found higher toxicity impacts in the second generation of *D. magna* individuals than in first-generation daphnids (of unexposed females) that occurred in 10 out of 19 compounds. Our data also showed the aggravated impact of CPZ on the observed parameters of population growth and reproduction success of *C. dubia* in subsequent F2 and F3 generations (Fig. 3); however, all mother daphnids in the F1 generation were exposed to study chemicals.

The only reference data on the effects of diclofenac on *C. dubia* were those from Ferrari et al. (2003). The authors have estimated the impact on 7-day-reproduction as NOEC = 1 mg/L and LOEC = 2 mg/L. In our study, the concentration of 1 mg/L had no significant effect on the population growth and reproduction success of each of the three successive generations during 30 days of observation, thus coinciding with the above reference data. Within this period, an inhibition of population growth became evident at 10 mg/L of DCF; however, only in the F2 and F3 generations. Noticeably more data on the DCF impact are available on *Daphnia magna* in the EnviroTox database (Connors et al., 2022). Acute toxicity to *D. magna* and *C. dubia* has been determined by Ferrari et al. (2003): 48-h EC50 values were 22.4 and 22.7 mg/L, respectively. Lesser sensitivity of this endpoint for *D. magna* has also been found in the range of 39.9–123 mg/L DCF (Cleuvers, 2004; Han, Hur & Kim, 2006; Haap, Triebskorn & Köhler, 2008; Ra et al., 2008; Lee et al., 2011; Quinn et al., 2011; Oliveira et al., 2015), which includes the 21-days LC50 = 56.6 mg/L as well (Quinn et al., 2011). Prolonged exposures of *D. magna* to DCF were shown to yield 21-day NOEC values of 10 mg/L (Quinn et al., 2011) and 25 mg/L (Lee et al., 2011); the former value corresponds to that for *C. dubia* found in the present study since survival was not affected at the highest tested DCF concentration of 10 mg/L in F1 generation.

Regarding the DCF effect on the reproduction of *D. magna*, 21-day NOEC has been reported to range from 8.3 to 21.3 mg/L (Cleuvers, 2004; Han, Hur & Kim, 2006; Lee et al., 2011), and this is approximately one order of magnitude higher than that to *C. dubia, i.e.*, 7-d NOEC = 1 mg/L, (Ferrari et al., 2003). Our data correspond with the latter reference result since DCF did not induce an inhibiting effect on population growth and reproduction success in the F1 generation of *C. dubia* at 0.1–10 mg/L. Although sensitivities of the two species to chemicals were reported to be generally similar (Versteeg et al., 1997; Connors et al., 2022), there was noticed by Connors et al. (2022) that several compounds composed an exception, including DCF with *D. magna/C. dubia* ratio = 18.

As in the case of CPZ, some stimulation of reproduction parameters of *C. dubia* was observed on day 21 but not on day 30 in the F1 and F3 at 1.0 mg/L of DCF. This indicates that the hormesis effect on reproduction traits is transient. It has been earlier reported (Dietrich et al., 2010) that the environmental concentration of DCF (0.36 µg/L) increased the offspring number per *D. magna* female in the F1 generation (equivalent to the F2 in our study). Further, the authors have found that the concentration used delayed age at first reproduction of *D. magna* in the F0 and F2 generations (equivalent to the F1 and F3 in our study). In contrast, we found that *C. dubia* of the F1 generation exposed to 1.0 mg/L DCF matured significantly faster than control daphnids (Supplementary Material). This somewhat contradictory data may reflect differences in daphnia species and thus various multigenerational effect consequences, which must be reliably investigated.

Data on lanthanides’ long-term toxicity to daphnids are relatively scarce in the scientific literature (Malhotra et al., 2020). As for gadolinium, Blinova et al. (2018) have reported 21-d LC50 = 0.49 mgGd/L for *D. magna*. In comparison, the highest concentrations of each lanthanide tested in our study were much more toxic to *C. dubia*, inducing 100% mortality within two and four days of exposure to Gd (0.425 mg/L) and Eu (0.41 mg/L), respectively (Supplementary Material). Further, the same laboratory (Blinova et al., 2022) has reported that 39-day exposure of *D. magna* to 0.1 mgGd/L had no adverse effect on the vitality, size and reproduction of parent animals and their offspring. Our data revealed that, within 21–30 days, a lower concentration of 0.0425 mgGd/L induced 30–40% mortality of *C. dubia* in the F1 generation and was lethal to 80% in the F3 generation. Based on the lethality data comparison, it can be noticed that *C. dubia* is more sensitive to tested lanthanides than *D.magna*. This is consistent with the data reported to DCF (Connors et al., 2022).

Different reactions to environmental toxicants depending on the daphnia generation found in our study have also been previously reported (Dalla Bona et al., 2016; Gosset et al., 2018). Castro et al. (2018) have pointed out that experimentation with one *D. magna* generation within 21 days of observation is not enough to fully reveal the effects of the representatives of four chemical classes. For instance, the impacts of copper sulphate and pharmaceutical paracetamol have become observable in the F2 generation. Similarly, our data confirm the absence of toxicological effects in the F1 generation of *C. dubia* exposed to tested concentrations of selected pharmaceuticals and lanthanides. Consequently, it is necessary to conduct long-term observations with several generations to obtain reliable information on possible adverse effects of environmental toxicants.

## Conclusions

A multigenerational study on *C. dubia* survival, population growth and reproduction success was implemented to assess the effects of representatives of two toxicant classes, pharmaceuticals and lanthanides. This study is the first attempt to investigate multigenerational effects on *C. dubia* exposed to chlorpromazine, diclofenac, and the chlorides of gadolinium and europium at close to environmentally relevant concentrations. In general, the data revealed a higher impact of all four chemicals in generations F2 and F3 than in F1, the first generation of treated daphnids; however, specific traits could be seen within the groups of chemicals.

No significant effect of the psychotropic drug CPZ on *C. dubia* survival was observed in generations F1–F3 during the 21-day exposure at 0.001–0.1 mg/L. Considerable stimulation of population growth and reproduction success took place at 0.001–0.01 mg/L in the F1 generation, while inhibition occurred at 0.1 mg/L in generations F2 and F3. Anti-inflammatory drug DCF did not affect survival in the F1 generation during 21-day exposure at 0.1–10 mg/L and considerably reduced survival in the F3 generation at 1–10 mg/L. As in the case of CPZ, (minor) stimulation of daphnids reproduction in the F1 generation and inhibition of population growth in generations F2 and F3 at 10 mg/L were traits of the DCF effect.

Both lanthanides did not affect survival in the F1 generation of *C. dubia* during 21-day exposure at 0.4 µg/L. A considerable decrease in survival at 4–42 µg/L was accompanied by significant inhibition of population growth and reproduction success of daphnids in the F2 generation for Gd and generations F2 and F3 for Eu. The inhibitory effect on the reproduction success of lanthanides in the F2 generation resembles that for CPZ but not for DCF. The use of *C. dubia* for indicating lanthanides’ effect seems promising due to its high sensitivity towards rear earths and since the environmental concentrations of REEs tend to increase above the contemporary measured *ca* 1 µg/L.

The dynamics of adverse effects during the 21–30-day period revealed that despite increased mortality in the controls (up to 30%), concentrations used in the study minified, in most instances, the survival and aggravated population growth and reproduction success of *C. dubia* significantly. Considering the longevity of *C. dubia,* 30%-mortality in control seems to be a reasonable level in exposures longer than 21 days. ISO standards require the mortality of the parent daphnids in the control not to exceed 20% at the end of the test, *i.e.,* 7–8 days for *C. dubia* and 21 days for *D. magna*. Our data suggest that *C. dubia* as a test organism can be used for 21 days in multigenerational investigations, especially when testing close to environmental concentrations. In this respect, the standard *C. dubia* chronic assay seems too short to serve as a basis for the prognosis of long-term harmful consequences. To summarise, prolonged observations throughout several generations of daphnids are required to obtain reliable information for the risk assessment of potentially aggressive chemicals.

##  Supplemental Information

10.7717/peerj.16472/supp-1 Data S1 Primary data of 40-day observationof *Ceriodaphnia dubia*, mean ± SD of the replicates in the control and treatments, brood number and days for the first brood

## Additional Information and Declarations

### Data Availability

The following information was supplied regarding data availability: The raw data are available in the Supplemental File.
